# Supplementary material for: Social distancing compliance: A video observational analysis
Source: PLoS One. 2021 Mar 15;16(3):e0248221. doi: 10.1371/journal.pone.0248221 (PMC7959357; doi:10.1371/journal.pone.0248221)
Supplement: S1 Appendix — (PDF) [file pone.0248221.s001.pdf]

# S1 Appendix: Codebook

Table S1.1. Codebook for the manual coding of CCTV-clips

| Code | Categories | Definition or explanation |
|---|---|---|
| Camera ID | | ID of the CCTV camera |
| Date | | Date of the observation |
| Type[ab] | D = Distance < 1.5 meter | Proximity: Two people find themselves within 1.5 meter of each other in public space (not in buildings or on vehicles) |
| | G = Group formation | Group formation: Three or more people form a group (i.e., all stand or sit together closely in public space) |
| Family/household | 0 = no;<br>1 = yes | Physical touching (e.g., holding hands) |
| Time | Hour:minute:second | First moment when the violation was observed |
| Duration of violation | 1 = 1 sec;<br>2 = 2-5 sec;<br>3 = more than 5 sec | |
| Nr. of people | | Number of people counted in still pictures at 5 different moments per clip |

[a] If two or more people pass two or more people, this was coded as D if this resulted in only two people being too close. If they were all in too close proximity, however, this was counted as G.

[b] The same people may engage in multiple incidents. For example, two people walk closely (within 1.5-meter) and then pass someone else (within 1.5-meter) or form a group with other people. In this case, it was coded as multiple incidents. However, if people are in close proximity, then distant themselves but eventually get close again, this was coded as one incident.
