# Supplementary material for: Social distancing compliance: A video observational analysis
Source: PLoS One. 2021 Mar 15;16(3):e0248221. doi: 10.1371/journal.pone.0248221 (PMC7959357; doi:10.1371/journal.pone.0248221)
Supplement: S2 Appendix — (PDF) [file pone.0248221.s002.pdf]

S2 Appendix:

Coded violations and number of people on the street with unimputed data

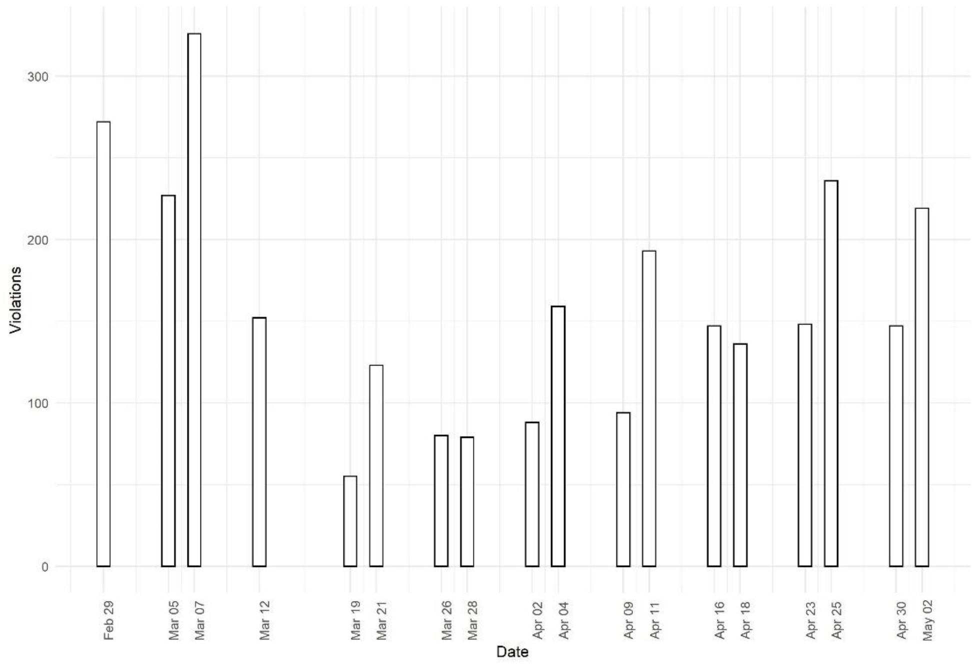

Fig S2.1. Observed social distancing violations (i.e., < 1.5 meter proximity by non-household members), unimputed data

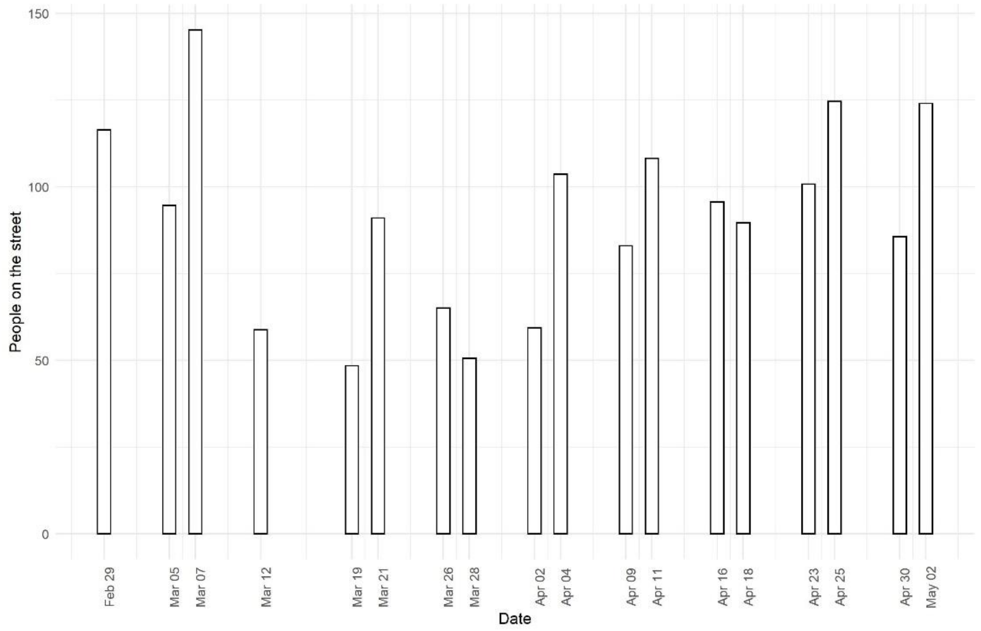

Fig S2.2. Number of people on the street, unimputed data
