# Supplementary material for: Social distancing compliance: A video observational analysis
Source: PLoS One. 2021 Mar 15;16(3):e0248221. doi: 10.1371/journal.pone.0248221 (PMC7959357; doi:10.1371/journal.pone.0248221)
Supplement: S3 Appendix — (PDF) [file pone.0248221.s003.pdf]

S3 Appendix:

Time series analyses

To further investigate the relations between observed social distancing violations and the nine contextual variables, we quantified their development over time and the relations between them. Our data represent a collection of ten potentially related time series, i.e., observations of values over ordered points in time. Despite the short length of the series (18 time-points) and the irregular intervals between time points (two days between Thursdays and Saturdays, and five days between Saturdays and Thursdays), some tools of time series analysis are useful to describe the features of the univariate time series and their correlations.

The autocorrelation function (ACF) describes the correlation between a variable measured at time t and the same variable measured at a previous time point t-k, where k is the spatial lag order. Fig S3.1 presents the autocorrelation functions of all ten time series. Although for some series more than 18 time points were available, to maximize comparability and subsequent comparisons between series, we only used the 18 time points that were available for all ten series. To ensure a minimum of 8 observations per time series, the lag function was limited to 10 lags (covering 5 weeks). As demonstrated in Fig S3.1, most series display a pattern whereby autocorrelations are relatively high at small lags and decrease as the time lags increase. This indicates that the values at recent prior time points are generally better predictors of the value at the next time point than values at points further back in time. This holds in particular for variables that measure the spread of the virus (i.e., the incidences of new COVID-19 deaths and transmissions), and to some extent also for frequencies of media items, for Google search scores, for temperature, and for the Google mobility data. The measures of social distancing violations and the number of people on the street display very weak autocorrelations.

To explore the relations between the observed numbers of social distancing violations and the other nine variables, we display cross-correlation functions (CCF) in Fig S3.2. The CCF is similar to the ACF, but it applies to two variables. It indicates the correlation between a measure of variable X at time *t+k* (*k* is the lag) and a measure of variable Y at time *t*. At *t=0*, it represents the correlation between X and Y at the same time point. For example, in Fig S3.2B, the correlation at lag = -10 represents the correlation between social distancing violations at time *t* and the temperature at time *t-10* (5 weeks ago). The value at lag = 10 is the correlation with the temperature at *t+10* (5 weeks later). The findings displayed in Fig S3.2 suggest that the medium to weak cross-correlations are affected by common linear trends and periodicity in the time series, where the periodicity reflects the Thursday-Saturday differential that is evident in some of the univariate time series (in particular numbers of media items published, but also the number of people on the street and social distancing violations).

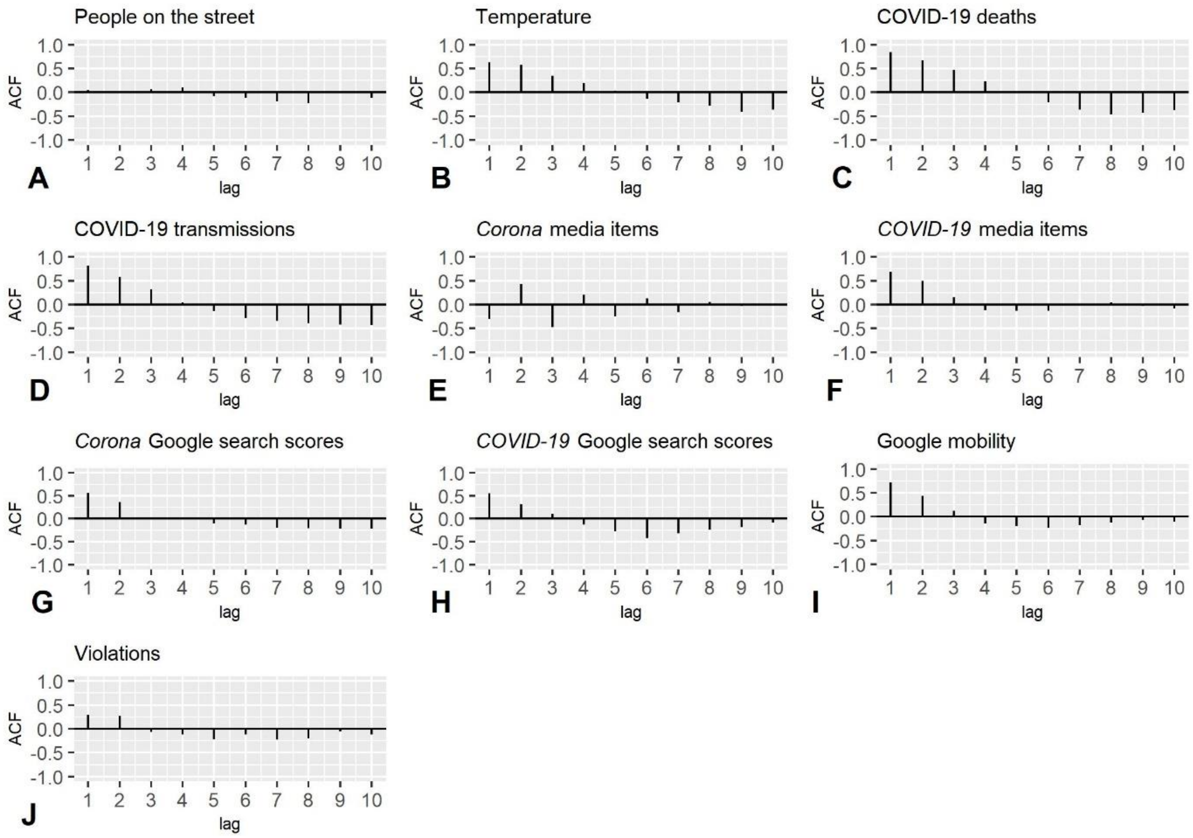

Fig S3.1. Autocorrelation function (ACF) of ten variables.

Time points are Thursdays and Saturdays between Saturday February 29th, 2020 and Saturday May 2nd, 2020.

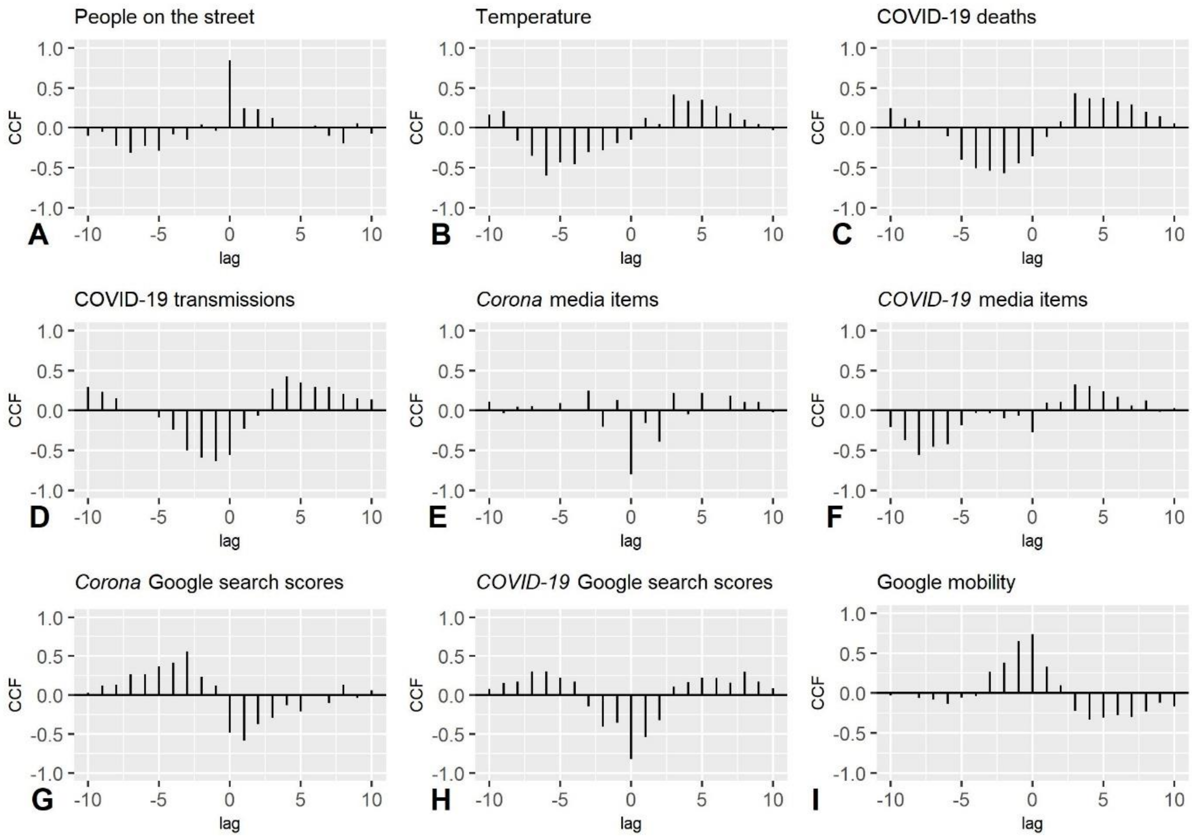

Fig S3.2. Cross-correlation function (CCF) of the number of violations with each of the nine other variables.

The estimated correlations are between social distancing violations at time $t + k$ and the other variable at time t, where k is the lag. Time points are Thursdays and Saturdays between Saturday February 29th, 2020 and Saturday May 2nd, 2020.
