# Supplementary material for: Social distancing compliance: A video observational analysis
Source: PLoS One. 2021 Mar 15;16(3):e0248221. doi: 10.1371/journal.pone.0248221 (PMC7959357; doi:10.1371/journal.pone.0248221)
Supplement: S4 Appendix — (PDF) [file pone.0248221.s004.pdf]

S4 Appendix:

Bivariate correlations and trivariate regression models with raw data

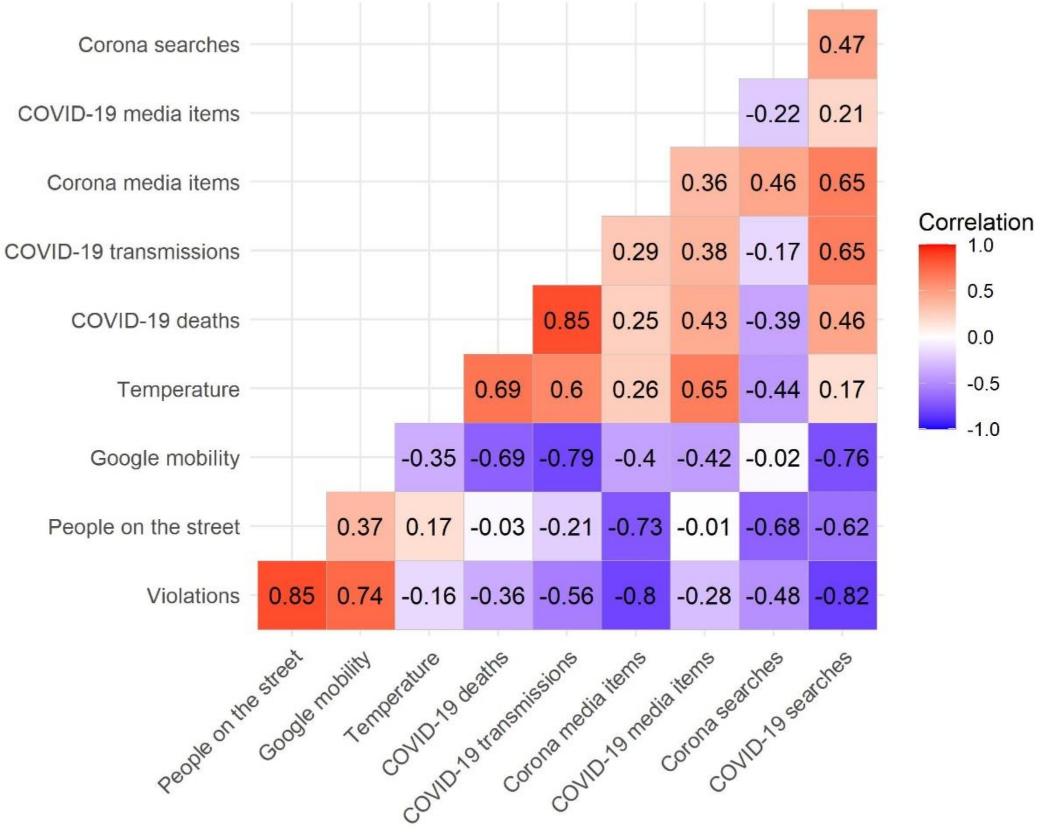

Fig S4.1. Correlation coefficients between variables, raw data

Table S4.1. Number of social distancing violations regressed on the number of people on the street and each of the other variables

	**Model 1**	**Model 2**	**Model 3**	**Model 4**	**Model 5**	**Model 6**	**Model 7**	**Model 8**
Intercept	55.92	-16.98	-25.22	4.73	71.21	-28.15	-110.66	95.89
	(27.07)	(34.72)	(31.04)	(30.46)	(66.01)	(35.70)	(65.21)	(47.59)
People on the	1.85***	2.52***	2.34***	2.13***	1.56**	2.36***	2.69***	1.52***
street	(0.22)	(0.32)	(0.30)	(0.27)	(0.49)	(0.34)	(0.51)	(0.35)
Google	1.76***							
mobility	(0.28)							
Temperature		-4.63*						
		(1.72)						
COVID-19			-0.37**					
deaths			(0.12)					
COVID-19				-0.06**				
transmissions				(0.02)				
Corona					-0.36*			
media items					(0.16)			
COVID-19						-3.71*		
media items						(1.69)		
Corona							0.65	
Google scores							(0.70)	
COVID-19								-1.44**
Google scores								(0.37)
R^2	0.92	0.81	0.83	0.86	0.79	0.78	0.73	0.86
N	18	18	18	18	18	18	18	18

***$p < 0.001$, **$p < 0.01$, *$p < 0.05$

Estimates from trivariate OLS regression models. Standard errors in parentheses.
