# Supplementary material for: Social distancing compliance: A video observational analysis
Source: PLoS One. 2021 Mar 15;16(3):e0248221. doi: 10.1371/journal.pone.0248221 (PMC7959357; doi:10.1371/journal.pone.0248221)
Supplement: S5 Appendix — (PDF) [file pone.0248221.s005.pdf]

S5 Appendix:

Detrending procedure

To assess the contemporaneous relationships between the time series of the social distancing violations and those of the other variables, we followed the approach typically advocated in the literature [41]. According to this approach, each time series is separately detrended by regressing it on a polynomial function of time and additional variables to account for cyclical time patterns (i.e., periodicity or ‘seasonality’) and subsequently using the residuals to assess relationships between the variables.

In our data, weekly periodicity between Thursday and Saturday measures is plausible for all measures that are dependent on routine activity behaviors, which typically differ between workday and weekends. Even reported COVID-19 deaths and transmissions depend to some extent on the day of the week, and temperature is likely the only variable to be unaffected by weekly cycles.

The choice of the polynomial time function is more complicated. There are competing arguments for increasing or limiting the order of the polynomial function. When third-order or higher-order terms are included the risk increases that the parameter estimates become overly dependent on coincidental outliers (‘overfitting’), in particular if the time series is short (N = 18 observations in this study). The general advice is to have the polynomial include not more than a quadratic or possibly a cubic term.

For each of the ten time-series, we estimated the following three ordinary least squares regression models:

(1) $Y = ß_0 S + u$

(2) $Y = ß_0S + ß_1T + u$

(3) $Y = ß_0S + ß_1T + ß_2T^2 + u$

(4) $Y = ß_0S + ß_1T + ß_2T^2 + ß_3T^3 + u$

where *Y* is the time series (e.g., people on the street), *S* is a dummy indicating Saturday (as opposed to Thursday), *T* is time measured in days since the start of the series on February 29, 2020, *u* is the residual, and the *ß* are parameters. Because the parameter estimates are not relevant to the detrending operation, we summarize the results of these analyses in Table S5.1 by reporting R^2 and the Bayesian Information Criterion (BIC) as indicators of model fit, and *T*-tests and likelihood ratio tests to test fit of each model against a more restricted version (e.g., Model 3 against the more restricted Model 2). Model 1 was tested against an intercept-only model.

The results on the BIC (for which lower values indicate better fit), *F*-test and likelihood ratio test (for which *p* values below .05 or .01 indicate a better fit on the less restricted model) are in agreement and suggest that for 5 variables (Temperature, *Corona* Google search scores, *COVID-19* Google search scores, COVID-19 transmission and COVID-19 deaths) Model 4 is preferred and for the other variables Model 3 is preferred. However, given the limited improvement in R^2 in Model 4 relative to in Model 3, and to reduce the risk of 'overfitting' associated with Model 4 (which would imply estimating a four-variable regression equation on only 18 data points), we decided to detrend all 10 variables using Model 3.

Table S5.1. Summary statistics of 10 variables each regressed on 4 sets of trend variables. *F*-tests and Likelihood ratio tests compare the fit of each model with that of the preceding model, and the fit of Model 1 with an intercept-only model. Lowest BIC scores printed bold.

	R^2				**Bayesian Information Criterion**				***F*-test**				**Likelihood ratio test**			
Model	1	2	3	4	1	2	3	4	1	2	3	4	1	2	3	4
Violations	0.27	0.32	0.72	0.76	206.21	207.77	**194.82**	195.00	0.03	0.30	0.00	0.17	0.02	0.25	0.00	0.10
Temperature	0.02	0.51	0.53	0.73	114.29	104.70	106.88	**99.83**	0.61	0.00	0.47	0.01	0.58	0.00	0.40	0.00
People on the street	0.32	0.34	0.56	0.60	167.96	170.35	**165.93**	166.98	0.02	0.53	0.02	0.26	0.01	0.48	0.01	0.17
Corona Google search	0.06	0.25	0.43	0.70	162.35	161.16	159.28	**150.28**	0.33	0.07	0.06	0.00	0.30	0.04	0.03	0.00
COVID-19 Google search	0.09	0.18	0.70	0.79	170.88	171.92	156.57	**153.35**	0.22	0.22	0.00	0.04	0.18	0.17	0.00	0.01
Corona media items	0.71	0.75	0.86	0.87	193.66	193.96	**185.65**	187.25	0.00	0.15	0.00	0.34	0.00	0.11	0.00	0.26
COVID-19 media items	0.04	0.56	0.56	0.59	116.02	104.83	**107.65**	109.28	0.41	0.00	0.82	0.35	0.38	0.00	0.79	0.26
COVID-19 transmissions	0.00	0.30	0.83	0.91	279.26	275.62	252.91	**245.00**	0.97	0.02	0.00	0.01	0.97	0.01	0.00	0.00
COVID-19 deaths	0.00	0.54	0.76	0.90	208.82	197.82	189.17	**175.77**	0.94	0.00	0.00	0.00	0.93	0.00	0.00	0.00
Google mobility	0.00	0.44	0.77	0.81	165.88	158.51	**144.99**	145.12	0.85	0.00	0.00	0.17	0.84	0.00	0.00	0.10

Model 1: Weekly Periodicity ($Y = \beta_0 S + u$)

Model 2: Weekly Periodicity + Linear Time ($Y = \beta_0 S + \beta_1 T + u$)

Model 3: Weekly Periodicity + Linear Time + Quadratic Time ($Y = \beta_0 S + \beta_1 T + \beta_2 T^2 + u$)

Model 4: Weekly Periodicity + Linear Time + Quadratic Time + Cubic Time ($Y = \beta_0 S + \beta_1 T + \beta_2 T^2 + \beta_3 T^3 + u$)
