# Supplementary material for: Social distancing compliance: A video observational analysis
Source: PLoS One. 2021 Mar 15;16(3):e0248221. doi: 10.1371/journal.pone.0248221 (PMC7959357; doi:10.1371/journal.pone.0248221)
Supplement: Attachment — Submitted filename: Response to Reviewers20-09-11.docx [file pone.0248221.s006.docx]

*Dear Editor,*

*We thank you for the opportunity to revise and resubmit our manuscript. We appreciate the feedback from the reviewers and have done our best to incorporate their suggestions into the revised draft. We feel the manuscript has improved as a result.*

*In the remainder of this memo, we explain how we have addressed the points raised by the academic editor and reviewers.*

*With kind regards,*

*The authors*

**Comments from the Editor**

Both of the reviewers have indicated concerns with the approach taken to analyse the data and the possible limitations in the data. It is important, that these concerns are addressed and if needed, a statistician sought to assist with updating the analysis.

*In response to the reviewers’ comments, we have conducted more rigorous additional analyses. We also explain why detrended data are needed.*

**Comments from Reviewer #1**

Overall, I was excited to read this paper that uses video data to measure social distancing compliance. This is an important source of data to complement the more prevalent uses of cell phone data.

*We thank the reviewer for their careful consideration of our work.*

I have the following concerns/comments:

1. The authors should clarify how 1.5m is measured in the videos. It is well known that a lot of media images showing beaches are misleading because of the focal points --- is this an issue here?

*While we acknowledge that our distance measures are not perfect, we would like to stress that the CCTV clips were collected in dense urban environments where various attributes were available that could serve as reference objects for the estimation of length. Coders are familiar with, for example, the size of sidewalk tiles, street furniture (e.g., benches, trash cans), and vehicles (e.g., bikes), which were readily available on the recordings both near and further away from the camera. Therefore, the assessment of the distance between people on the clips used in this study is likely to be more accurate than assessments following from (media) images of beaches or abandoned parking lots that the reviewer appears to refer to.*

*To address the reviewer’s comment, we added a note to the manuscript (page 7) that makes readers aware of the potential effect of focal length of cameras on our distance measures.*

2. It looks like most of the change in 1.5m compliance occurred prior to the national mandate. This is consistent with previous work showing the importance of voluntary measures in the US. E.g.,
- Chetty, Raj, John N. Friedman, Nathaniel Hendren, and Michael Stepner. 2020. Real-Time Economics: A New Platform to Track the Impacts of COVID-19 on People, Businesses, and
Communities Using Private Sector Data.
- Allcott, Hunt, Levi Boxell, Jacob Conway, Billy Ferguson, Matthew Gentzkow, and Benny Goldman.
2020. Economic and Health Impacts of Social Distancing Policies during the Coronavirus Pandemic.

In general, it would also be good to make sure the literature review is up to date when published.

*We added a comment on the importance of voluntary action to the conclusion (page 22), added citations to the suggested papers, and conducted a new literature search to ensure our manuscript is up-to-date. We added several references as a result of this literature search (Brezinski et al., 2020; Chu et al., 2020; de Moraes, 2020; Ghader et al., 2020; Islam et al., 2020; Nguyen et al., 2020a; 2020b; Seale et al., 2020).*

3. In a lot of the correlations, the authors detrend the data. I'm not sure that is what you want to do. E.g., When examining the relationship between covid cases and social distancing behaviors, most people are going to be making inferences about the trends --- not deviations from those trends. It would be good to visualize the correlations both ways.

*We visualize and interpret the time-trends in the various variables (e.g., covid-19 cases, social distancing violations) based on the raw data. As advocated in the literature on time series (e.g., Farnum and Stanton, 1989, cited in revised manuscript), we use detrended variables when assessing the relationships between variables, to account for autocorrelation and common dependence on time.*

*In response to this comment, we elaborated on the necessity of detrending data for assessing relationships between two or more time series on page 11.*

4. I'd maybe suggest removing the time series analysis section and placing it in a supplementary appendix. It's hard to know what inferences to make from these. I'd be interested in seeing a multivariate regression analysis looking at what predicts violations (e.g., cases, deaths, temperature, media mentions, etc).

*We moved the time series analyses section (autocorrelation functions and cross-correlation functions) to the supporting material (Appendix S3) and conducted trivariate regression analyses (presented in Table 2, findings are discussed on page 20). To integrate the findings from the regression analyses into the paper, we rewrote parts of the conclusion and moved some text from the conclusion to the introduction section.*

5. I'm a bit concerned that the rise in 1.5m violations is purely mechanically related to the number of people in the video. I think it is important to try to distinguish between (a) 1.5m violations increasing purely because the # of people out is increasing vs (b) 1.5 m violations increasing because people are being less careful conditional on being outside. The correlation plot suggests a lot of this could be driven by (a).

Two ways I can think of addressing this:
- Compare the relationship between 1.5m violations and distancing in a placebo time peirod (e.g., a month or two before Covid or during the same period in 2019).
- Compute the expected number of violations if people were placed randomly in the video screen. (This isn't ideal, but could still be suggestive of how much of this is mechanical.)

*We agree that there is a 'mechanical' (i.e., geometric) relation between the number of people present and the distances they can stay away from each other. However, the relation with distances actually observed is not trivial. To account for the situational constraint that the number of people on the street places on social distancing compliance, we included the number of people as a covariate in a new set of trivariate regression analyses. See our response to the previous comment, as well as Table 2 in the main document and the description of our findings on page 20.*

*In addition, to better distinguish between 'compliance with stay-at-home directives' on the one hand and 'people present on the street' on the other hand (also in response to comment #7 of Reviewer #1), we added analyses using large-scale data from Google Mobility about the number of visitors at non-residential locations in Amsterdam, based on cellphone location information.*

*We thank the reviewer for suggesting alternatives. However, the first suggestion would require data that are not available. As for the second suggestion, we believe that random placement would not provide a valid baseline for human movement in space (e.g., people cluster in groups, follow walking lines based on physical attributes of the location). Instead, we decided in favor of the solution described above (trivariate regression and adding Google Mobility data).*

Other points not required for revision, but would be great to see addressed if possible:
6. I was disappointed with the limited scope of the data actually analyzed. I was hoping machine learning was used to identify violations which would have allowed a minute-by-minute overview of these patterns across time and much richer analysis. The study as-is is still useful, but not to the same degree.

*In the revised manuscript, we list promising technologies, including machine learning, to support automated identification of social distancing violations, and provide references to the relevant literature (page 27).*

7. It would be great to compare the video violations to cell phone distancing behavior --- since that was part of the motivation for the paper.

*Good point! In response, we included comparisons with Google Mobility data, which are based on aggregated cellphone location information.*

8. It would be nice if the violations were also coded by demographics. E.g., Were 50% of all people on the video male, but males composed 70% of the violations?

*We agree with the reviewer that this would be an interesting addition to our study. However, this would require us to re-do the coding for all the footage to not only assess the gender of the people in the video, but also to assess the two-party issue (i.e., whether violations were between only males, only females, or males and females). We currently do not have the manpower to conduct this additional round of coding and, since it is not part of our research question, have decided to refrain from doing so.*

**Comments from Reviewer #2**

This paper studies an important and timely question: examining whether citizens are adhering to social distancing practice set by the government, and whether such adherence last over time is critical to inform how we project the effectiveness of such policies. This study uses a novel measure of social distancing behaviors from CCTV camera footage in Amsterdam, a measure that is rare in the existing literature.

*We thank the reviewer for their evaluation of our manuscript.*

With this said, I think the paper’s analyses have a number of important limitations.

1. The authors do not perform any rigorous statistical tests, and hence it is impossible to tell whether there indeed is an upward trend of social distancing violation over time. With the current number of observations in the analyses, I do not think the authors have sufficient statistical power to draw any conclusions.

*In the previous version of the manuscript, we indeed only presented a description of the time trend in social distancing violations time based on a visual inspection of the data.*

*In response to the reviewers’ comment, we now also present the findings of a multivariate regression with the number of social distancing violations regressed on linear time, quadratic time, and periodicity (i.e., Thursday versus Saturday), see Table 1 and the discussion of these findings on pages 13-14. The findings show that the quadratic model, demonstrating a U-shaped pattern, provides a much better fit to the data than the linear model. Thus, even with the limited number of observations in this study, we can detect a non-linear trend in the number of social distancing violations over time. Specifically, we observe that the number of violations decreased between February 29^th^ and early April, and that this number increased from early April to early May.*

2. The primary outcome of interest is the total level of social distancing violation. This is very difficult to interpret, since as the authors show, the total number of people shown on the street is also increasing over time. Hence, it is not clear whether the rate of social distancing violation actually increases, which I believe is what the authors ultimately are interested in. Moreover, I do not think the current evidence can substantiate the claim that social distancing compliance and stay-at-home compliance coincide. One could mechanically result in the other: the absolute number of social distancing violation would go up (even if the compliance rate remains constant) if people are less likely to stay at home.

*To be sure, we are not ultimately interested in the rate (violations by the number of people on the street). We are interested in time-trends and correlates of the number of social distancing violations.*

*That said, in working on this revision we realized that our measure of the number of people on the street offers a better representation of ‘crowdedness’ than of ‘compliance to stay-at-home directives’. Therefore, and in response to comment #7 of Reviewer #1, we decided to conduct additional analyses with data from Google Mobility about the number of visitors at various locations in Amsterdam based on cellphone information.*

*Further, in response to this comment and to comments from Reviewer #1, we have added trivariate regression analyses in which the relationship between social distancing violations on the one hand and potential explanatory factors on the other hand (i.e., mobility, temperature, new covid-19 deaths, new covid-19 transmissions, media items, and internet searches) are assessed while controlling in each regression equation for the number of people on the street. The findings show that compliance to stay-at-home directives (as captured with Google Mobility data) is related to social distancing violations even after correcting for the number of people on the street.*
